# The symptoms of autism including social communication deficits and repetitive and restricted behaviors are associated with different emotional and behavioral problems

**DOI:** 10.1038/s41598-020-76292-y

**Published:** 2020-11-25

**Authors:** Ching-Hong Tsai, Kuan-Lin Chen, Hsing-Jung Li, Kuan-Hsu Chen, Chao-Wei Hsu, Chun-Hsiung Lu, Kuan-Ying Hsieh, Chien-Yu Huang

**Affiliations:** 1 Department of Child & Adolescent Psychiatry, Kaohsiung Municipal Kai-Syuan Psychiatric Hospital, No. 130, Kaisyuan 2nd. Rd., Lingya Dist., Kaohsiung City, 802 Taiwan (R.O.C.); 2 Department of Occupational Therapy, College of Medicine, National Cheng Kung University, Tainan City, Taiwan (R.O.C.); 3 Department of Physical Medicine and Rehabilitation, National Cheng Kung University Hospital, College of Medicine, National Cheng Kung University, No. 1, University Rd., Tainan City, 701 Taiwan (R.O.C.); 4 Institute of Allied Health Sciences, College of Medicine, National Cheng Kung University, Tainan, Taiwan, No. 1, University Rd., Tainan City, 701 Taiwan (R.O.C.); 5 School of Occupational Therapy, College of Medicine, I-Shou University, No. 8, Yida Rd., Kaohsiung City, 82445 Taiwan (R.O.C.)

**Keywords:** Health care, Signs and symptoms

## Abstract

The aim of the study was to investigate the associations between symptoms of autism and emotional and behavioral problems in children with autism spectrum disorder from both caregivers’ and clinicians’ perspectives. Three main findings were found in our study. First, the association patterns were similar in the preschool and school-aged children. Second, different association patterns were found from caregivers’ and professionals’ perspectives. From the professionals’ perspective, only repetitive behaviors were associated with behavioral problems, while from the caregivers’ perspective, all symptoms of autism were associated with emotional and behavioral problems. Third, different types of symptoms of autism were associated with different types of emotional and behavioral problems. For example, from the professionals’ perspective, restricted and repetitive behaviors were only associated with hyperactivity and inattention. From the caregivers’ perspective, social emotion was associated with emotional symptoms, and other symptoms of autism were associated with hyperactivity and inattention, as well as with peer problems. The results of our study provided deeper understanding of the relationships between symptoms of autism and emotional and behavioral problems, and the findings could serve as a reference for intervention planning when clinicians approach children with autism spectrum disorder.

## Introduction

Children with autistic spectrum disorders (ASD) are characterized by two main symptoms: deficits in social interaction and communication, and restricted and repetitive behaviors^1^. In addition to these inherent symptoms of autism, epidemiological studies have shown that children with ASD have a high prevalence of presenting emotional and behavioral problems (EBPs), such as anxiety, depression, hyperactivity and inatten, and aggressive behaviors^2–8^. It is thus important to identify whether the two symptoms of autism are respectively related to specific EBPs. Identifying their associations could further guide clinicians for intervention planning by taking into account the two variables together when approaching children with ASD.

A number of studies have investigated the effects of symptoms of autism on EBPs in the ASD population. Based on the types of EBPs, the results can be viewed from two aspects, emotional symptoms (e.g., depression and anxiety, or internalizing behaviors) and behavioral problems (e.g., aggression and challenging behaviors). The results of the relationships between symptoms of autism and emotional symptoms have been inconsistent. Lindsey et al. conducted a longitudinal study to investigate the interactions between autism symptom severity and parenting behaviors in predicting change in child behavior problems^9^. They recruited 129 children with ASD aged 4–10 years. The results showed that ASD symptom severity was significantly and positively correlated with both internalizing and externalizing problems at the initial and follow-up time points. In contrast, Mazurek et al. investigated friendship and internalizing symptoms (anxiety/depression) among 1,202 children and adolescents with ASD^10^. The results showed that greater ASD severity was associated with fewer symptoms of anxiety/depression, lower IQ, and poorer number and/or quality of reciprocal friendships. Sterling et al. investigated characteristics associated with depressive symptoms in 46 adults with ASD^11^. They found that individuals with less social impairment, higher cognitive ability, and higher rates of other psychiatric symptoms were more likely to report depressive symptoms.

Moreover, two studies by Kim et al. and Strang et al. found no significant associations between symptoms of autism and depressive symptoms^4,12^. Kim et al. specifically investigated anxiety and mood problems in 59 children with high functioning ASD or Asperger syndrome. They found that the number of psychiatric problems was not correlated with early symptoms of autism but that psychiatric problems were predicted by early verbal/non-verbal IQ discrepancy scores^4^. Strang et al. also examined depression and anxiety symptoms in children and adolescents with ASD without intellectual disability. The results showed that higher IQ or fewer ASD symptoms were not found among individuals with both ASD and depression or anxiety symptoms^12^. Since there have been no conclusive results regarding the effects of symptoms of autism on emotional symptoms, more studies are warranted.

In contrast, the effects of symptoms of autism on behavioral problems are consistently positive. Studies by Mastson et al. and Jang et al. both found that challenging behaviors were positively associated with symptoms of autism^6,13^. Kanne et al. investigated 1,380 children and adolescents aged from 4 to 17 years^3^. They found that aggressive behaviors were more likely to be related to increases in repetitive behaviors, higher income, and increasing levels of ASD-related social and communicative deficits^3^. Toisika et al. found that, in comparison to the findings for children with intellectual disability, only hyperactivity was significantly higher in children with ASD and intellectual disability^14^. Moreover, after controlling for intellectual disability and maternal mental health, the presence of ASD significantly increased the odds for hyperactivity, conduct problems and emotional symptoms. Also, as mentioned above, Lindsey et al. found that ASD symptom severity was significantly and positively correlated with child externalizing problems^9^. In summary, children with greater severity of symptoms of autism are likely to have more behavioral problems.

Although a number of studies have discussed the relationships between symptoms of autism and EBPs, two critical issues need to be discussed. First, the age ranges of the sample subjects were too broad or too narrow. Those with broad age ranges covered children to adolescents (e.g., 6–16 years^15^, 4–17 years^3^, or 2–18 years^13^), and one narrow study focused only on 5-year-old children in a cross-sectional study^11^. It is known that children in different developmental stages have their own characteristics. Children in different developmental stages may present different EBPs, such as emotional symptoms in preschool children and conduct problems in school-aged children. When all children and adolescents are grouped together, it is hard to distinguish whether the results are contributed by the children, the adolescents, or both. Furthermore, narrow age range of study participants interferes the generalization of results to other children with age bands. It has been suggested that more homogeneous age grouping (e.g., only focusing on children) be adopted to clarify the associations clearly and concisely for better clinical application.

Second, few studies have simultaneously considered two perspectives (professionals and caregivers) to investigate symptoms of autism. For example, the studies of Lindsey et al.^9^, Toisika et al.^14^, and Jang et al.^13^ used a parent report questionnaire to assess symptoms of autism, while the studies of Strang et al.^12^, Mazurek et al.^10^, Matson et al.^6^, Sterling et al.^11^, and Kim et al.^4^ used a professional-administered assessment to identify symptoms of autism. Only the study by Kanne et al.^3^, considered two perspectives. Symptoms of autism can be assessed by professionals (e.g., with the CARS, the Autism Diagnostic Interview, and the Autism Diagnostic Observation Schedule [ADOS]) or by caregivers (e.g., with the SRS, the Autism Spectrum Disorder Behavior Checklist [ASD-BC], and the Repetitive Behavior Scale-Revised). However, professionals and caregivers may perceive children’s symptoms of autism differently^16^, which may cause different results on the associations. For example, the studies by Strang et al. and by Samon et al. both used the Child Behavior Checklist to assess emotional symptoms (anxiety/depression and emotional dysregulation)^12,15^, while Strang et al. used the ADOS (a professional report through caregiver interview)^12^ and Samon et al. used the ASD-BC (parent-report) to assess symptoms of autism^15^. Significant associations between emotional symptoms and symptoms of autism were found in Samon et al.’s study but not in Strang et al.’s study. Therefore, to comprehensively identify the associations between symptoms of autism and EBPs, the perspectives of both professionals and caregivers should be considered.

Consequently, the present study aimed to investigate the associations between symptoms of autism and EBPs in children with ASD. We specifically focused on children aged 3–12 years, who were separated into a preschool group and a school-aged group, and used two measures, i.e., the CARS and the SRS™-2, to assess symptoms of autism from the perspectives of professionals and caregivers.

## Methods

### Participants

Children with ASD were recruited from five hospitals and pediatric rehabilitation clinics. The inclusion criteria were (1) diagnosis of ASD, and (2) age from 3 to 12 years old. Caregivers who could not communicate in Mandarin or read Chinese were excluded from the study.

### Measures

#### Childhood Autism Rating Scale (CARS)

The CARS^17^, a 15-item scale for children aged above two years old, was used to assess symptoms of autism. Each item of the CARS is rated on a 4-point scale ranging from 1 (normal) to 4 (severely abnormal). Children were categorized into three severity levels according to the total score. A cutoff score of 25.5 has been suggested to be most accurate for identifying ASD in children with full-scale intelligence quotients (IQs) of 80 or higher^18^. In our study, two cut-off points were adopted to identify ASD; the cut-off points of 30 and 25.5 were respectively used for children with IQs of below 80 and those with IQs of 80 or higher. Considering the child’s evaluation compliance, we used the verbal comprehension index (VCI) of the Wechsler Preschool and Primary Scale of Intelligence™—Fourth Edition (WPPSI-IV) and the VCI of the Wechsler Intelligence Scale for Children—Fourth Edition (WISC-IV) as references for IQ. For the Chinese sample, Cronbach’s *α* coefficient of the CARS was 0.735. Fifteen items of the CARS showed significance for differential diagnosis of autism (*p* < 0.01). The correlations between the total score of the CARS and its own 15 items ranged from 0.569 to 0.935. The coefficients between the 15 items ranged from 0.278 to 0.808, and the correlation between the total score of the CARS and the autism behavior checklist was 0.502^19^.

To examine the associations of symptoms of autism with EBPs, we adopted the 2-factor model of the CARS suggested in Park and Kim’s study: social communication and interaction, and restricted and repetitive behaviors^20^. The sum scores of each factor (referred to as the subscale scores) were used in the study.

#### The Social Responsiveness Scale™, Second Edition (SRS™-2)

The school-age version of the SRS™-2 is a 65-item caregiver- and/or teacher-report measure assessing social functioning in children^21^. Unlike the original version of the SRS™-2, the Chinese version of the SRS™-2 has a four-factor structure: general features, autistic mannerisms, social awareness, and social emotion^22^. We adopted the four-factor structure to examine their relationships with EBPs. The Chinese version of the SRS™-2 has good test–retest reliability (intraclass correlations = 0.75–0.85) and internal consistency (Cronbach’s *α* = 0.94–0.95)^22^.

#### Strengths and Difficulties Questionnaire-Chinese version (SDQ)

The Strengths and Difficulties Questionnaire-Chinese version (SDQ) was used to assess EBPs in children with ASD in our study. The SDQ contains five domains, with four assessing difficulties (emotional symptoms, hyperactivity/inattention, conduct problems, and peer relationship problems) and one assessing a strength (prosocial behaviors). Each domain has 5 items scored on a 3-point rating scale, and the sum of the items in each domain generates a domain score. A higher score in these four difficulty domains represents a higher number of problems, while a higher score in the strength domain indicates fewer problems. Each domain categorizes children’s problems into three severity levels: close to average, at risk, and at high risk of having emotional or behavioral problems. The Chinese SDQ showed good test–retest reliability (intraclass correlations = 0.71–0.85), satisfactory internal consistency (Cronbach’s α = 0.40–0.86), satisfactory concurrent validity with the child behavior checklist, and good discriminant validity (significantly discriminant attention deficit/hyperactivity disorder and non-attention deficit/hyperactivity disorder)^23^.

### Procedures

Cover letters explaining the research project and consent forms were distributed to caregivers referred by clinicians. The researchers contacted caregivers who agreed to participate in the study, and they executed the following steps. First, a researcher conducted a semi-structured interview with the caregiver to administer the CARS. Meanwhile, the researcher also observed the child’s behaviors to confirm with the caregiver their responses to the CARS items. Second, the researcher assessed the child’s VCI with the WISC-IV or WPPSI-IV, depending on the child’s age. Third, while the researcher assessed the child, the caregiver completed a demographic questionnaire, the SDQ and the SRS™-2. Completed questionnaires were returned to researchers. Questionnaires were checked for missing data, and missing data were collected by phone calls to the caregivers.

### Statistical analysis

Descriptive analyses were conducted to characterize the children’s demographics, symptoms of autism, and EBPs. We separated the children into two age groups, preschool children (the preschool group, 3 to 6 years) and school-aged children (the school-aged group, 7 to 12 years), and the following analyses were respective conducted on the two groups. Pearson correlation analyses were used to examine the correlations within subscales of the CARS and the SRS™-2, as well as the correlations of the SDQ with the CARS and with the SRS™-2.

Multiple stepwise regression models were separately conducted to examine the effects of symptoms of autism (i.e., the CARS or the SRS™-2) on the EBPs (i.e., the SDQ), controlling for children’s demographic characteristics and cognitive function. The five SDQ subscale scores were the dependent variables. The independent variables were children’s age and gender, VCI score, and the subscale scores of the CARS or SRS™-2. All the significance levels were set at *α* = 0.05. All analyses were conducted in SPSS 18.0 software.

### Ethical approval

This study was approved by Institutional Review Boards of Kaohsiung Municipal Kai-Syuan Psychiatric Hospital and Chi-Mei hospital in Taiwan. All methods in your study were carried out in accordance with relevant guidelines and legislation.

### Informed consent

Informed consent was obtained from all individual participants included in the study.

## Results

### Characteristics of participants

Table 1 shows the characteristics of the participants. A total of 108 children with ASD were recruited in the study, with 58 preschool (mean age = 65.67 months, SD = 12.49) and 50 school-aged children (mean age = 99.28 months, SD = 21.54).

**Table 1:** Characteristics of the participants (N = 108).

Variables	Statistics	
	Preschool group (n = 58)	School-aged group (n = 50)
Age (months): mean (SD)	65.67(12.49)	99.28 (21.54)
Sex (male): n, %	49 (84.5)	45 (90.0)
**CARS: mean (SD)**		
Social communication and interaction	10.55(1.63)	10.60 (1.50)
Restricted and repetitive behaviors	18.88 (2.41)	20.52 (2.72)
Total scores	29.40 (3.34)	31.12 (3.76)
**SRS™-2:**		
General features	37.76 (14.00)	42 (15.12)
Autistic mannerisms	19.33 (6.36)	20.72 (7.73)
Social awareness	19.31 (3.98)	20.38 (4.63)
Social emotion	11.72 (4.08)	12.00 (4.18)
Total score	88.12 (24.10)	95.10 (28.15)
**Verbal Comprehension Index: n, %**		
Below 80	8 (13.8)	7 (14.0)
90 > VCI ≥ 80	4 (6.9)	9 (18.0)
110 > VCI ≥ 90	20 (34.5)	19 (38.0)
Above 110	36 (44.8)	15 (30.0)
**Strength and Difficulties: means, SD**		
Emotional symptoms	3.66 (2.14)	3.94 (2.43)
Conduct problems	2.60 (1.75)	2.86 (1.68)
Hyperactivity and inattention	7.05 (2.43)	6.70 (2.48)
Peer relationship problems	5.24 (1.81)	5.18 (1.92)
Prosocial behaviors	4.79 (2.29)	5.16 (2.12)
Total difficulties	18.55 (5.32)	18.68 (6.16)

CARS: Childhood Autism Rating Scale, SRS™-2: Social Responsiveness Scale.

For the two groups, the two subscales of the CARS had moderate correlation (*r* = 0.36 and 0.55 respectively in the preschool group and school-aged group) with each other. Among the SRS™-2 subscales, general features and autistic mannerisms had high correlations (*r* = 0.90 and 0.93 respectively in group 1 and group 2). The subscales of general features and autistic mannerisms both had high correlations with the subscale of social emotion (*r* = 0.77–0.79), but low to moderate correlations with the subscale of social awareness (*r* = 0.13 and 0.47 respectively in group 1 and group 2). Table 2 shows the correlations within the CARS and the SRS™-2.

**Table 2:** The correlations of the five subscales of the SDQ with the subscales of both the CARS and the SRS™-2. (N = 108).

CARS		Social interaction		Restricted and repetitive behaviors
**Preschool group**				
Social interaction		–		0.36**
**School-aged group**				
Social interaction		–		0.55**

SRS™-2	General features	Autistic mannerisms	Social awareness	Social emotion
**Preschool group**				
General features	–	0.90**	0.12	0.78**
Autistic mannerisms	–	–	0.12	0.77**
Social awareness	–	–	–	0.13
**School-aged group**				
General features	–	0.93**	0.38**	0.79**
Autistic mannerisms	–	–	0.37**	0.77**
Social awareness	–	–	–	0.47**

*p < *.*05; **p < *.*01, CARS: Childhood Autism Rating Scale, SRS™-2: Social Responsiveness Scale.

### The relationships between the EBPs and symptoms of autism

Table 3 shows the correlations of the SDQ with the CARS and with the SRS™-2 in the two groups. In both groups, the CARS subscale of social interaction had no significant associations with the five SDQ subscales, except the subscale of hyperactivity and inattention in the school-aged group (*r* = 0.56). The CARS subscale of restricted and repetitive behaviors had small to moderate correlations with the subscales of hyperactivity and inattention (*r* = 0.42 and 0.31 respectively in the preschool and school-aged groups) and peer relationship problems (*r* = 0.26 and 0.28 respectively in the preschool and school-aged groups).

**Table 3:** The correlations of the five subscales of the SDQ with the subscales of both the CARS and the SRS™-2. (N = 108).

Group	CARS		SRS™-2			
	Social interaction	Restricted and repetitive behaviors	General features	Autistic mannerisms	Social awareness	Socialemotion
**Preschool group**						
Emotional symptoms	−0.07	0.17	0.3*	0.26*	−0.11	0.40**
Conduct problems	0.07	0.22	0.25	0.24	0.05	0.20
Hyperactivity and inattention	0.24	0.42**	0.53**	0.43**	0.34**	0.36**
Peer relationship problems	0.10	0.26*	0.52**	0.41**	0.08	0.32*
Prosocial behaviors	−0.19	0.02	−0.10	−0.08	−0.32*	−0.06
**School-aged group**						
Emotional symptoms	0.17	0.01	0.46**	0.46**	0.34*	0.61**
Conduct problems	0.15	0.18	0.32*	0.34*	0.30*	0.37**
Hyperactivity and inattention	0.56**	0.31*	0.43**	0.47**	0.53**	0.42**
Peer relationship problems	0.25	0.28*	0.60**	0.62**	0.28	0.43**
Prosocial behaviors	−0.13	0.08	−0.17	−0.16	−0.42**	−0.23

*p < *.*05; **p < *.*01, CARS: Childhood Autism Rating Scale, SRS™-2: Social Responsiveness Scale.

As for the SRS™-2, the subscales of general features and autistic mannerisms had similar correlational patterns with the subscales of the SDQ in the two groups. These two subscales had small to moderate correlations with the subscales of emotional symptoms (*r* = 0.26–0.46), hyperactivity and inattention (*r* = 0.43–0.53), and peer relationship problems (*r* = 0.41–0.62). Moreover, general features/autistic mannerisms also had significant correlations with conduct problems in the school-aged group (*r* = 0.32-0.34). The subscale of social awareness revealed different patterns from those of the five SDQ subscales. In the preschool group, the subscale of social awareness was only associated with hyperactivity and inattention (r = 0.34) and prosocial behaviors (*r* = -0.32), but in the school-aged group, the subscale of social awareness was associated with all the SDQ subscales (*r* = -0.42–0.53) except that of peer relationship problems (*r* = 0.28). In both groups, the subscale of social emotion was associated with the subscales of emotional symptoms (*r* = 0.40–0.61), hyperactivity and inattention (*r* = − 0.36–0.42), and peer relationship problems (*r* = 0.32–0.43). Moreover, in the school-aged group, the subscale of social emotion was also associated with conduct problems (*r* = 0.37).

### The effects of symptoms of autism on the EBPs

Table 4 summarizes the effects of the symptoms of autism on the EBPs in the two groups. We first investigated the effects of the two CARS subscales respectively on the five SDQ subscales. In the two groups, of the five SDQ subscale models, only one was significant; i.e., the subscale of hyperactivity and inattention. After controlling for the children’s verbal comprehension ability, age, sex, and social interaction, the subscale of restricted and repetitive behaviors was significantly and positively associated with the SDQ subscale of hyperactivity and inattention (standardized β = 0.45 and 0.54 respectively in the preschool and school-aged groups).

**Table 4:** Summary of the predictions of symptoms of autism on the emotional and behavioral problems. (N =108).

Group	CARS		SRS™-2		
	Social interaction	Restricted and repetitive behaviors	General features/Autistic mannerisms	Social awareness	Social emotion
**Preschool group**					
Emotional symptoms	X	X	X	X	+ (standardizedβ = 0.53)
Conduct problems	X	X	X	X	X
Hyperactivity and inattention	X	+ (standardizedβ = 0.45)	+ (standardizedβ = 0.61)	+ (standardizedβ = 0.27)	X
Peer relationship problems	X	X	+ (standardizedβ = 0.84)	X	(standardizedβ = −0.40)
Prosocial behaviors	X	X	X	X	X
**School-aged group**					
Emotional symptoms	X	X	X	X	+ (standardizedβ = 0.55)
Conduct problems	X	X	X	X	X
Hyperactivity and inattention	X	+ (standardizedβ = 0.54)	X	+ (standardizedβ = 0.50)	X
Peer relationship problems	X	X	+ (standardizedβ = 0.54)	X	X
Prosocial behaviors	X	X	X	X	X

X: no significant relationship, +: positive significant relationship, −: negative significant relationship.

Because the two SRS™-2 subscales, i.e., general features and autistic mannerisms, had high correlation with each other (*r* = 0.92), we only included the subscale score of the general features as an independent variable to prevent collinearity between the variables in the regression models. The two groups showed similar patterns, and the five SDQ subscale models had different contributors. The models of emotional symptoms were significantly and positively associated with social emotion (standardized ß = 0.53 and 0.55 respectively in the preschool and school-aged groups) in the two groups. The model of conduct problems was significant, but it was significantly associated only with verbal comprehension and not with any of the subscales of the SRS™-2. The model of hyperactivity and inattention was significantly associated with general features (standardized ß = 0.61) and social awareness (standardized ß = 0.27) in the preschool group and significantly associated only with social awareness (standardized ß = 0.50) in the school-aged group. The model of peer problems was associated with general features (standardized ß = 0.84) and social emotion (standardized ß = − 0.40) in the preschool group and with general features (standardized ß = 0.54) in the school-aged group. The models of prosocial behaviors were not significant in either group.

## Discussion

The study aimed to investigate the associations between symptoms of autism and EBPs in children with ASD. There were three main findings. First, generally, similar correlation patterns between the symptoms of autism and EBPs were revealed for the two age groups. Second, the symptoms of autism assessed by professionals and by caregivers showed different association patterns with the EBPs. Third, the two symptoms (deficits in social communication and interaction, and restricted and repetitive behaviors) were respectively associated with specific EBPs.

It is interesting to note that in the CARS, the subscale of social communication and interaction had no significant effects on any of the SDQ subscales, and the subscale of restricted and repetitive behaviors was only associated with the subscale of hyperactivity and inattention. This result was consistent with a study by Gabriels et al., which investigated the relationship between repetitive behaviors and associated clinical features (e.g., other behavior problems)^24^. When nonverbal cognitive ability, adaptive level, and sleep problems were controlled for, they found a significant positive correlation between the presence of repetitive behaviors and the hyperactivity scale of the Aberrant Behavior Checklist.

The subscale of hyperactivity and inattention was related to the general autistic features of the SRS-^TM2^ in preschool children and by the social awareness scale of the SRS-^TM2^ in both preschool and school-aged children. It is reasonable that hyperactivity and inattention was associated with general features, since numerous studies have identified overlapping features between the two diagnoses of ASD and ADHD. A study by Rommelse, Franke, Geurts, Hartman, & Buitelaar showed that ADHD and ASD share behavioral, neuropsychological and neurobiological characteristics^25^. The two disorders co-occur with high frequency, for 20–50% of children with ADHD meet the criteria for ASD and 30–80% of children with ASD meet the criteria for ADHD. Our results are also similar to those of a study by Russell, Rodgers, and Ford, which showed that hyperactivity scores were particularly high in children with ASD^26^.

We found that, from the caregivers’ perspective, children’s social awareness was positively associated with hyperactivity. This result was consistent with previous studies. For example, a study by Factor et al. investigated whether the presence of anxiety and ADHD symptoms added to social impairment in children with ASD^27^. They found that children with heightened ADHD traits showed higher scores on two subscales of social communication and social awareness. Also, a study by Hoza found that deficits in social knowledge and social immaturity are often linked to ADHD^28^.

The results showed that the general features of the SRS™-2 contributed to peer relationship problems. In the SDQ, peer relationship problems are described as children having no friends, being less popular, and getting bullied. Children with greater severity had more problems in social communication and interaction, and they had more restricted and repetitive behaviors. Those difficulties can cause peer problems. Moreover, social emotion was found to be negatively associated with peer problems in the regression model. However, when we investigated the univariate correlation analysis, the correlation between social emotion and peer problems was positive. The conflicting results may have resulted from the high correlation of the subscales of social emotion and general features in our study. They may have shared the same explained variance of the peer problems when both variables were entered in the regression model, which may have caused the coefficient of social emotion to turn from positive to negative.

It is noted that the social emotion subscale of the SRS™-2 was associated with the emotional symptoms of the SDQ in our study, while previous studies produced no consistent results on the relationships between emotional symptoms and symptoms of autism. A possible reason may be that the social emotion and emotional symptoms assessed in our study measured related concepts. Social emotion indicated the emotional aspects of behaviors in the social context (e.g., item 10, ‘‘literally’’, and item 46, ‘‘facial expressions’’). It is highly possible that children who do not exhibit proper emotional behaviors in social contexts also present emotional symptoms, such as tantrums or depression.

A few limitations were noted in this study. First, to decrease the test burden on the children and caregivers, increase their compliance, and achieve test accuracy, the measures used in our study tended to be short (e.g., the SDQ and the CARS) or easy to administer (e.g., the SRS-2, and the VCI of the WPPSI-IV and the WISC-IV) but have sound psychometric properties. This study can be viewed as a preliminary study to identify the relationships between symptoms of autism and EBPs. More comprehensive assessments of the EBPs (e.g., the child behavior checklist), the symptoms of autism (e.g., the ADOS or the ADI-R), and the cognitive function (the full scales of the WPPSI-IV and the WISC-IV) should be conducted to clarify the associations in depth or to have better test control. Second, according to the results of the CARS, most children recruited in our study had mild to moderate ASD. The results of our study thus may not be generalizable to children with severe ASD. Last, a cross-sectional design, not a longitudinal design, was adopted in our study, so the cause–effect relationships between the two variables could not be identified. Future studies adopting a longitudinal design could focus on the significant associations found in our study to identify the corresponding cause–effect relationships.

## Conclusion

This study aimed to investigate the associations between symptoms of autism and EBPs by considering both clinicians and caregivers’ perspectives and by separating the children into two different age groups. The results revealed that the symptoms of autism perceived by caregivers and clinicians showed different association patterns with EBPs, and that different symptoms of autism were associated with specific EBPs. The results could serve as a reference for intervention planning to help clinicians pay more attention to specific EBPs when approaching children with ASD.

## What is Known

- Symptoms of autism were positively associated with emotional symptoms and behavioral problems.

## What is New

- Different association patterns were found between symptoms of autism and emotional and behavioral problems when different assessments of the symptoms of autism were used.
- The perspectives of caregivers and clinicians on children’s symptoms of autism were quite different.

## Data Availability

The data of this study are available to Editorial Board Members and referees.
